# The Pleasure Evoked by Sad Music Is Mediated by Feelings of Being Moved

**DOI:** 10.3389/fpsyg.2017.00439

**Published:** 2017-03-21

**Authors:** Jonna K. Vuoskoski, Tuomas Eerola

**Affiliations:** ^1^Faculty of Music, University of OxfordOxford, UK; ^2^Department of Music, University of JyväskyläJyväskylä, Finland; ^3^Department of Music, Durham UniversityDurham, UK

**Keywords:** sad music, being moved, music-induced emotion, empathy, liking, beauty

## Abstract

Why do we enjoy listening to music that makes us sad? This question has puzzled music psychologists for decades, but the paradox of “pleasurable sadness” remains to be solved. Recent findings from a study investigating the enjoyment of sad films suggest that the positive relationship between felt sadness and enjoyment might be explained by feelings of being moved (Hanich et al., 2014). The aim of the present study was to investigate whether feelings of being moved also mediated the enjoyment of sad music. In Experiment 1, 308 participants listened to five sad music excerpts and rated their liking and felt emotions. A multilevel mediation analysis revealed that the initial positive relationship between liking and felt sadness (*r* = 0.22) was fully mediated by feelings of being moved. Experiment 2 explored the interconnections of perceived sadness, beauty, and movingness in 27 short music excerpts that represented independently varying levels of sadness and beauty. Two multilevel mediation analyses were carried out to test competing hypotheses: (A) that movingness mediates the effect of perceived sadness on liking, or (B) that perceived beauty mediates the effect of sadness on liking. Stronger support was obtained for Hypothesis A. Our findings suggest that – similarly to the enjoyment of sad films – the aesthetic appreciation of sad music is mediated by being moved. We argue that felt sadness may contribute to the enjoyment of sad music by intensifying feelings of being moved.

## Introduction

Why do people sometimes enjoy listening to music that makes them sad? The paradox of “pleasurable sadness” has attracted significant research interest among music psychology scholars in recent years (for a review, see Sachs et al., 2015), but the puzzle remains to be solved. A body of empirical work has shown that listening to nominally sad music induces a multifaceted emotional response that is not clearly negative or positive (e.g., Vuoskoski et al., 2012; Kawakami et al., 2013; Taruffi and Koelsch, 2014), and that there are certain personality variables that are consistently associated with the enjoyment of sadness-inducing music (e.g., Garrido and Schubert, 2011; Vuoskoski et al., 2012; Taruffi and Koelsch, 2014; Eerola et al., 2016). Furthermore, sad music has been shown to induce sadness-related biases in memory and judgment (Vuoskoski and Eerola, 2012, 2015), suggesting that listening to sad music is indeed able to induce ‘genuine’ sad affective states in listeners.

During the past two decades, multiple theoretical accounts for the ‘sadness paradox’ have been proposed by different scholars. Schubert (1996) postulated that, although positive and negative emotions are typically connected to pleasure and displeasure (respectively), the connection between negative emotion and displeasure gets inhibited in an aesthetic context such as music listening. However, this account does not explain why certain nominally negative music-induced emotions such as sadness are enjoyed, while others such as fear are not (see Vuoskoski et al., 2012). Huron (2011) has further extended the idea that music-induced sadness is disconnected from the negative real-word implications and displeasure that are typically associated with experiences of sadness, and proposed that the pleasure sometimes experienced while listening to sad music might be related to the adaptive, consoling physiological responses (such as the release of prolactin) triggered by a sad affective state. While this account fits together nicely with empirical findings linking greater intensity of felt sadness with greater enjoyment (e.g., Vuoskoski et al., 2012; Eerola et al., 2016), direct empirical evidence for the potential role of prolactin and other hormones is currently still lacking. Moreover, Juslin (2013) has criticized the fact that the proposed prolactin/consolation effect is actually an ‘after-effect’ rather than a pleasurable experience of listening to sadness-inducing music, and that in this account “there is no ‘pleasurable sadness,’ there is only pleasure following sadness” (Juslin, 2013; p. 258).

Juslin (2013), on the other hand, has proposed that the enjoyment of sadness-inducing music might have nothing to do with sadness itself, but that sad music is experienced as pleasurable simply because it is aesthetically pleasing or ‘beautiful’. Indeed, empirical studies have documented strong positive correlations between perceived sadness and perceived beauty (at least in Western music tradition; Eerola and Vuoskoski, 2011), and some of the most intense aesthetic listening experiences have been brought about by sad music (Gabrielsson and Lindström, 1993; Eerola and Peltola, 2016). But rather than solving the paradox, Juslin’s proposal brings us back to the original problem: What exactly makes sad music so profoundly ‘beautiful,’ and is ‘beautiful’ not just another way of describing pleasurable stimuli?

The concept of ‘beauty’ is central to the aesthetic appreciation of music (Istók et al., 2009; Brattico and Pearce, 2013), although aesthetic experiences comprise other components as well. In the broader context of music-related aesthetic experiences, Brattico et al. (2013) propose distinguishing between aesthetic emotions (e.g., feelings of awe, enjoyment, and interest), aesthetic judgments (the appraisal of beauty, proficiency, and other aesthetic dimensions), and conscious liking (involving decisional, evaluative processes). They argue that conscious liking succeeds aesthetic emotions and judgments in the temporal domain, and might even occur independently of them. Preliminary empirical work suggests that conscious liking, perceived beauty and pleasantness are highly inter-correlated constructs (*r*s = 0.56–0.87; Eerola and Vuoskoski, 2011), but indeed not identical. Although significant advances have been made in uncovering the neural underpinnings of music-induced pleasure (including aesthetic ‘chills’; Blood and Zatorre, 2001; Salimpoor et al., 2011) and in distinguishing between the brain structures involved in liking and the perception of happiness and sadness (Brattico et al., 2016), the constituents of musical ‘beauty’ are not yet well understood (e.g., Brattico et al., 2013).

A new clue for the ‘sadness paradox’ may be offered by recent findings from the field of film studies. Hanich et al. (2014) investigated the enjoyment of sad films using 38 film clips as stimuli, and found that the initial positive relationship between felt sadness and enjoyment was entirely mediated by feelings of being moved. Experiences of ‘being moved’ are still not fully understood due to a lack of psychological research, but Menninghaus et al. (2015; see also Kuehnast et al., 2014) have recently offered a comprehensive account of the phenomenon: On the basis of multiple exploratory studies, they concluded that feelings of being moved are typically evoked by critical life and relationship events such as birth, death, and marriage, but also by exposure to artworks, nature, and music. Importantly, the two main emotional ingredients of being moved appear to be sadness and joy. The instances of joy and/or sadness that give rise to the special emergent feeling of being moved seem to have certain common characteristics: Events eliciting feelings of being moved are often characterized by high compatibility with prosocial norms and self-ideals, and the person experiencing the emotion is typically unable to affect the event or its outcome. Wassiliwizky et al. (2015) extended the findings of Hanich et al. (2014) by investigating emotional and aesthetic responses to sad and joyful film clips. In line with Hanich et al. (2014), they found that the positive relationship between felt sadness and enjoyment was entirely mediated by feelings of being moved, but no mediation effect was found for the relationship between felt joy and enjoyment. Crucially, they also found that participants often used empathy-related words such as “compassionate” and “sympathetic” to describe their emotional responses to the sad film clips, corroborating the link between sadly moving scenarios and prosocial norms and ideals (see Menninghaus et al., 2015). Indeed, Menninghaus et al. (2015) have proposed that feelings of being moved may serve a social bonding function by activating the value of social bonds and prosocial behavior, and thus those high in socially responsive traits such as empathy may be especially prone to experience feelings of being moved.

Interestingly, Wassiliwizky et al. (2015) also found that feelings of being moved were the best predictor of the likelihood of experiencing aesthetic chills; pleasurable bodily sensations commonly described as a ‘spreading gooseflesh’ (cf. Panksepp, 1995). Feelings of sadness were also positively associated with the likelihood of chills, but there was no statistically significant association between felt joy and chills. Similar findings have also been obtained in the context of music listening, where sad music has been found to be significantly more likely to evoke aesthetic chills than happy music (Panksepp, 1995). Although it has been more than a decade since Konecni (2005) outlined aesthetic awe, being moved, and chills as the three central aesthetic responses (awe being the rarest and most profound of the three, and chills being the most common), feelings of ‘being moved’ have rarely been explicitly studied in the context of music listening. A recent study by Eerola et al. (2016), however, explored the structure of emotions experienced in response to nominally sad, unfamiliar music, and found that feelings of being moved played a central role in enjoyable feelings of music-induced sadness. More specifically, they found that feelings of sadness, being moved, and liking all loaded strongly on the same latent emotion factor that they subsequently labeled ‘Moving sadness,’ and that experiences of ‘Moving sadness’ were significantly predicted by trait empathy. However, it is not yet known whether feelings of being moved might actually mediate the positive relationship between felt sadness and enjoyment as in the case of sad films (Hanich et al., 2014; Wassiliwizky et al., 2015).

### The Present Study

The aim of the present study was to investigate the hypothesis that feelings of being moved would mediate the positive effect of felt sadness on enjoyment in the context of music listening. Enjoyment was operationalized as ‘liking’ for the music. Furthermore, since trait empathy has been repeatedly implicated in the enjoyment of sad music (e.g., Garrido and Schubert, 2011; Vuoskoski et al., 2012; Taruffi and Koelsch, 2014; Eerola et al., 2016) and in the psychological phenomenon of being moved (Menninghaus et al., 2015; Wassiliwizky et al., 2015), it was hypothesized that trait empathy would contribute to feelings of being moved evoked by sad music. Experiment 1 explored the potential mediating role of ‘being moved’ and the contribution of trait empathy in an online listening setting. Experiment 2 was carried out in a laboratory, and was designed to untangle the complex relationships between perceived sadness, movingness, beauty, and liking.

## Experiment 1

### Method

#### Ethics Statement

The experimental protocol was approved by the Ethics Committee of the University of Jyväskylä, Finland. All participants gave their written, informed consent, and the study was carried out in accordance with the approved guidelines.

#### Participants

Three hundred and thirty-eight participants from different countries took part in an online experiment. After deleting partial answers and outliers (i.e., those participants whose inter-group correlation was 2 SDs below the mean inter-group correlation), we were left with 308 participants (239 female) aged 18–75 (*M* = 31.7, *SD* = 9.7). Participants were recruited by distributing the experiment link on social media (Facebook, Twitter, and Reddit). Two hundred two participants (61.2%) were Finnish, 10.6% were American, 5.2% British, 4.5% German, and 18.5% were other nationalities.

#### Stimulus Material

The stimuli were selected by a panel of five expert judges (including the authors). With the aim to find a variety of music examples that would successfully convey sadness, each panel member selected three musical examples from different genres. The resulting 15 music examples were then rated by all panel members using the same set of rating scales as used by the participants of the main experiment (see Procedure –section below). Out of these 15 music examples, four examples were chosen for the main experiment on the basis that they conveyed differing (yet sufficient) levels of sadness as well as varying degrees of movingness and other emotional qualia (e.g., peacefulness and anxiety). The four selected examples were *Oblivion* (composed by Astor Piazzolla, performed by Stjepan Hauser with the Zagreb Philharmonic Orchestra), *Darkness* (by Lacrimosa), *Something I Can Never Have* (by Nine Inch Nails), and *Together We Will Live Forever* (by Clint Mansell), representing different genres (classical, film music, and gothic and industrial rock). Two of the examples contained lyrics (*Darkness* and *Something I Can Never Have*). Furthermore, a fifth piece, *Discovery of the Camp* (composed by Michael Kamen), was included on the basis that it has successfully been used in previous studies to induce sadness and feelings of being moved (Vuoskoski and Eerola, 2012; Eerola et al., 2016). Two-minute excerpts of each of these five pieces were used as the stimuli in the experiment.

#### Measures

Two subscales of The Interpersonal Reactivity Index (IRI; Davis, 1983), Fantasy and Empathic Concern, were used to measure participants’ trait empathy. The IRI is a widely used, multifaceted measure of trait empathy, and the two aforementioned subscales have been repeatedly implicated in studies investigating individual differences in the enjoyment of sadness-inducing music (Garrido and Schubert, 2011; Vuoskoski et al., 2012; Eerola et al., 2016). We also included a measure of trait emotional contagion, The Emotional Contagion Scale (ECS; Doherty, 1997), since recent work has shown the ECS to be – in addition to Fantasy and Empathic Concern – one of the best predictors of the enjoyment of unfamiliar, sadness-inducing music (Eerola et al., 2016).

#### Procedure

The experiment was carried out online using the *Qualtrics*-platform. In order to minimize self-selection bias related to music-induced emotions (and sadness in particular), participants were recruited by promising them individualized feedback on their personality traits (based on their trait empathy scores). Participants were told that they would hear some music in the experiment, but emotions were not mentioned in the study advertisement. The five musical stimuli were presented in a random order, and the participants were instructed to listen to the entire excerpt before giving their ratings. They were asked to wear headphones if possible to ensure optimal sound quality. The experiment was programmed in such a way that the participants could not move to the next excerpt before 1 min had passed. The participants were asked to rate how much they liked each excerpt, and to describe their emotional reaction (‘How did you feel when you listened to the music?’) using seven adjective scales (*sad, melancholic, moved, in awe, peaceful, anxious*, and *powerless*). The selection of rating scales was based on a previous study that explored the underlying factor structure of emotional responses to sad-sounding music (Vuoskoski and Eerola, submitted), as the objective was to provide the participants with a selection of scales that would satisfactorily reflect the range and type of emotions typically experienced in response to sad music. The scale extremes were labeled “Does not describe my emotional reaction at all” and “Describes my emotional reaction very well.” The liking and emotion ratings were given using slider scales ranging from 0 to 100. The participants were also asked to rate the familiarity of the music excerpts (on a 4-point scale). After listening to all five music excerpts and reporting their felt emotions, liking, and familiarity, the participants filled in the trait empathy questionnaires.

### Results

#### Descriptive Statistics

**FIGURE 1:**
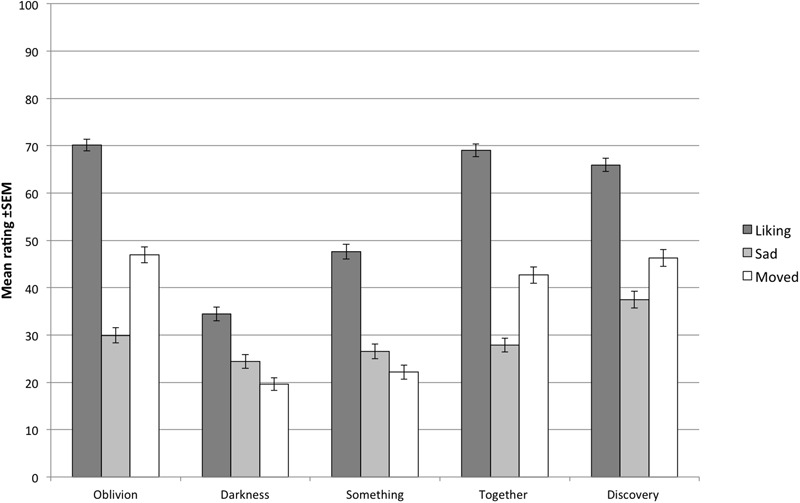
**The mean ratings (± standard error of the mean) of liking, felt sadness, and being moved given to the five musical excerpts**.

The mean familiarity ratings for the five music excerpts ranged from 1.13 to 1.60, (on a scale from 1 to 4) indicating that the excerpts were unfamiliar to the majority of participants. The mean ratings of liking, felt sadness and being moved given to the five music excerpts are displayed in **Figure 1**, demonstrating the variability of the felt emotions and liking responses evoked by the different stimuli. In order to explore the general pattern of associations among the ratings of felt emotion and liking, Pearson correlation coefficients were calculated for each participant using their raw ratings, and then averaged over participants (see **Table 1** for the correlation matrix). Because of the high number of correlations and the descriptive nature of the analysis, we have refrained from making inferences regarding the statistical significance of the correlations. The correlations revealed a strong positive association between liking and being moved (*r* = 0.69), a moderate correlation between felt sadness and being moved (*r* = 0.29), and a small correlation between liking and felt sadness (*r* = 0.22), thus providing grounds for a mediation analysis.

**Table 1:** Pearson correlations coefficients between ratings of felt emotion and liking.

	Liking	Sad	Moved	Melanc.	Anxious	Powerless	Peaceful
Sad	0.22						
Moved	0.69	0.29					
Melancholic	0.36	0.52	0.39				
Anxious	-0.29	0.24	-0.20	-0.03			
Powerless	-0.05	0.25	-0.01	0.19	0.24		
Peaceful	0.56	0.09	0.48	0.26	-0.36	-0.09	
In awe	0.52	0.11	0.55	0.21	-0.17	-0.04	0.36

*The correlations were first calculated within each participant, and then averaged across participants.*

#### Mediation Analysis

The hypothesis that the enjoyment of sadness-inducing music (i.e., the positive association between felt sadness and liking ratings) is mediated by feelings of being moved was tested through a multilevel (1–1–1) mediation analysis, following the method documented by Bauer et al. (2006). Essentially, this approach – also used by Hanich et al. (2014) – provides all the necessary information for evaluating the hypothesized causal effects of the mediation model by combining the dependent variable (liking) and the mediator (being moved) into a single stacked response variable, and running a mixed model with selection variables for the DV and mediator to toggle between models. Multilevel mediation analysis was used because of the structure of the rating data, which represented a nested structure with the five music excerpts (at Level 1) nested within participants at Level 2; the model included random slopes and random intercepts for participants. Confidence interval for the mediation (indirect) effect was calculated using the method presented by Preacher and Selig (2010). The analyses were carried out in R using the lme4-package (Bates et al., 2015).

**FIGURE 2:**
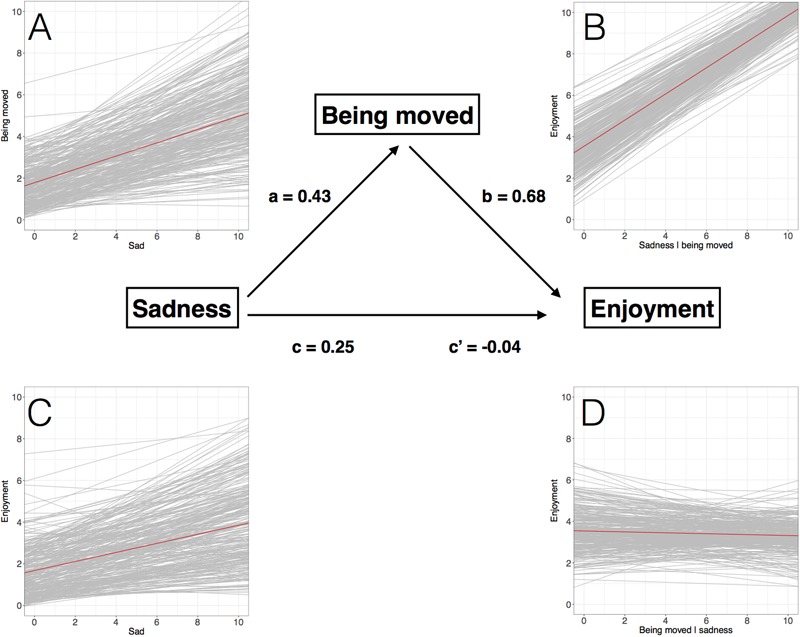
**The coefficients for the paths a, b, c, and c′ and the corresponding random-slope plots for the multilevel mediation analysis where enjoyment (liking) was the dependent variable, felt sadness was the independent variable, and being moved the mediator.** In the random-slope plots **(A–D)** the thin gray lines represent the individual slopes of the participants, while the red line represents the mean slope. Plot **(A)** visualizes the individual slopes for the effect of felt sadness on being moved; plot **(B)** for the effect of being moved on enjoyment conditioned on felt sadness; plot **(C)** for the total effect of felt sadness on enjoyment; and plot **(D)** for the effect of felt sadness on enjoyment conditioned on being moved.

The paths, coefficients, and random-slope plots of the multilevel mediation analysis are visualized in **Figure 2**. The total effect of felt sadness on liking was significant (path c; β = 0.25, *t* = 8.46). The effect of felt sadness on being moved (path a; β = 0.43, *t* = 13.82), and the effect of being moved on liking (path b; β = 0.68, *t* = 32.85) were also significant. However, when feelings of being moved were controlled for, the direct effect of felt sadness on liking became non-significant (path c′; β = -0.042, *t* = -1.67). The estimated indirect effect of felt sadness on liking (mediated by feelings of being moved) was 0.30 (95% CI [0.25; 0.34]), suggesting that the positive relationship between sadness and liking was entirely mediated by feelings of being moved.

If we adopt a broader view of felt sadness and include melancholy into an aggregate measure of felt sadness (felt sadness + felt melancholy), the pattern of coefficients in the mediation analysis remains relatively unchanged: The total effect of felt sadness on liking becomes somewhat stronger (path c; β = 0.38, *t* = 11.65) as does the effect of felt sadness on being moved (path a; β = 0.56, *t* = 18.41). The effect of being moved on liking remains very similar (path b; β = 0.62, *t* = 27.49), and when feelings of being moved are controlled for, the direct effect of felt sadness on liking becomes non-significant once again (path c′; β = 0.046, *t* = 1.58). The estimated indirect effect of felt sadness on liking was practically unchanged (0.33; 95% CI [0.29; 0.38]), further confirming that the positive effect of felt sadness on liking is mediated fully by feelings of being moved.

#### Individual Differences

Finally, we explored the hypothesis that trait empathy would contribute to feelings of being moved, as well as the possibility that trait empathy might modulate the relationships between felt sadness, being moved, and liking. Fantasy, Empathic Concern, and Emotional Contagion were all significantly correlated with mean ratings of felt sadness (*r*s = 0.18–0.23, *p* < 0.001–0.01) and being moved (all *r*s = 0.25, *p* < 0.001), but only Empathic Concern was significantly correlated with mean liking ratings (*r* = 0.15, *p* < 0.01). However, none of the trait empathy variables were significantly correlated with the individual slope coefficients extracted from the first multilevel mediation analysis, suggesting that – although trait empathy may contribute to the overall intensity of felt sadness and feelings of being moved (and thus liking) – it does not modulate the relationships between the variables.

### Discussion

In line with previous findings obtained using film clips (Hanich et al., 2014; Wassiliwizky et al., 2015), we found that the initial positive relationship between felt sadness and liking was entirely mediated by feelings of being moved. The results were almost identical regardless of the type of felt sadness used as the independent variable; ratings of felt sadness, or an aggregate of felt sadness and felt melancholy. The close similarities in the patterns of mediation obtained in the present study and in those by Hanich et al. (2014) and Wassiliwizky et al. (2015) are especially remarkable when the differences in the operationalization of ‘enjoyment’ are taken into consideration. In the present study, we used conscious liking as the dependent measure, while Hanich et al. (2014) and Wassiliwizky et al. (2015) used the degree of ‘wanting to see the entire film’ as a proxy for enjoyment (a decision for which they provide a well-argued explanation). Nevertheless, the closely replicated path coefficients in the mediation models suggest that both operationalizations seem to tap into the same broader construct of ‘enjoyment.’

As hypothesized, measures of trait empathy (Fantasy, Empathic Concern, and Emotional Contagion) were positively correlated with the mean ratings of being moved. Trait empathy was also associated with ratings of felt sadness, although only Empathic Concern was significantly correlated with mean liking ratings. These results corroborate the findings of previous studies (e.g., Garrido and Schubert, 2011; Vuoskoski et al., 2012; Taruffi and Koelsch, 2014; Kawakami and Katahira, 2015), most notably those of Eerola et al. (2016), who found that Fantasy, Empathic Concern, and Emotional Contagion were the best predictors of experiences of ‘Moving sadness.’ However, we did not find any association between trait empathy and the individual slope coefficients extracted from the multilevel mediation model, suggesting that trait empathy did not modulate the relationships between felt sadness, being moved, and liking.

The findings and conclusions of Experiment 1 are subject to certain limitations and considerations. First, as the experiment was carried out on an online platform, we did not have any control over the listening situation or the amount of attention that participants paid to the listening and rating tasks. Second, in order to prevent experiment fatigue and drop-outs, we only used a small number (5) of stimuli. This prevented any systematic variation of stimulus features, although the selected stimuli were deliberately intended to evoke varying degrees of liking and being moved. Thus, Experiment 2 was designed to address these limitations, and to further explore the hypothesized mediating role of being moved in the enjoyment of sad music.

## Experiment 2

The aim of Experiment 2 was to try and elucidate the interconnections of perceived sadness, movingness, beauty, and liking in a laboratory setting. Previous work has shown perceived sadness and beauty to be highly correlated (*r* = 0.59; Eerola and Vuoskoski, 2011), but it is not known whether this covariance is inherent to the two phenomena, or whether they just happen to be correlated in the Western music corpus. Furthermore, it is not fully understood what qualities or features contribute to perceived beauty in the context of music listening. We set out to select musical material where levels of sadness and beauty would vary as independently as possible, as this would enable us to investigate Juslin’s (2013, p. 258) claim that “It is not that the sadness *per se* is a source of pleasure, it only happens to occur together with a percept of beauty.” Specifically, we wanted to test two competing hypotheses: (A) that movingness mediates the effect of perceived sadness on liking, or (B) that perceived beauty mediates the effect of sadness on liking. We also wanted to investigate whether movingness might mediate the positive relationship between perceived sadness and beauty. The decision was made to focus on perceived rather than felt emotion, as emotion perception can be reliably measured using relatively short stimuli (see e.g., Eerola and Vuoskoski, 2011, 2012). The shorter duration allowed us to use a larger number (27) of stimuli, and thus systematically vary levels of perceived sadness and beauty.

### Method

#### Ethics Statement

The experimental protocol was approved by the Ethics Committee of the University of Jyväskylä, Finland. All participants gave their written, informed consent, and the study was carried out in accordance with the approved guidelines.

#### Participants

The participants of Experiment 2 were 19 music students from the University of Jyväskylä (studying musicology or music education) aged 20–45 (*M* = 24.74, *SD* = 5.50, 15 female).

#### Stimulus Material

The stimuli were 27 short film music excerpts (duration: 13–26 s; *M* = 17.56, *SD* = 3.27) that were selected from a pool of 403 excerpts with pre-existing ratings of perceived emotion (360 excerpts from Eerola and Vuoskoski, 2011, and 43 excerpts from an unpublished dataset; *n* = 9). Pre-existing ratings of perceived beauty were present for a subset of 110 excerpts from Eerola and Vuoskoski (2011), but not for the remaining excerpts. For these 293 excerpts, the beauty ratings were estimated using a regression model based on ratings of perceived emotion (built using the dataset of 110 excerpts from Eerola and Vuoskoski, 2011). Based on the actual and estimated mean ratings of perceived beauty and sadness, we selected 27 examples where levels of beauty and sadness would vary as independently as possible; low, medium, and high levels of both in a 3 × 3 factorial design; three excerpts per combination. In the selected set of stimuli, 24 excerpts were from the set of Eerola and Vuoskoski (2011), while three excerpts were from the unpublished set (see Supplementary Material for the list of excerpts).

#### Procedure

The experiments were conducted individually for each participant using customized software built in the MAX/MSP graphical programming environment (version 5.1), running on Mac OS X. The excerpts were presented in a different random order to each participant. Because of the relatively high number and short duration of the musical stimuli, participants were asked to rate perceived (i.e., what the music sounds like) rather than felt emotion. The participants were asked to describe their perceived emotions using six adjective scales (range: 0–100); *sad/melancholic, moving/touching, tender/warm, peaceful/relaxing, scary/distressing*, and *happy/joyful* (translated from Finnish by the first author); the scale extremes were “Does not describe the music at all”, and “Describes the music very well.” Participants were also asked to rate how much they liked each excerpt, and how beautiful it sounded. Participants listened to the excerpts through studio quality headphones, and were able to adjust the sound volume according to their own preferences.

### Results

#### Descriptive Statistics

The mean ratings of perceived sadness and beauty for the different types of excerpts are displayed in **Table 2**. Note that the excerpts have been categorized according to the mean ratings obtained in the present experiment (three excerpts per category) rather than those used in the stimulus selection process. The mean ratings highlight the difficulty of finding film music excerpts – even from a database of over 400 examples – that would be both highly sad and low in perceived beauty. We further explored the covariance between perceived emotions, liking, and beauty by calculating Pearson correlation coefficients using the raw ratings of participants.

**Table 2:** Mean ratings (and standard deviations) of perceived beauty and sadness for the nine different types of excerpts (scale range: 0–100).

	Low sadness		Medium sadness		High sadness	
	*Beauty*	*Sadness*	*Beauty*	*Sadness*	*Beauty*	*Sadness*
Low beauty	32.2 (23.6)	22.7 (26.6)	50.2 (22.4)	50.8 (30.9)	65.7 (21.7)	68.3 (26.4)
Medium beauty	63.6 (23.7)	15.8 (21.9)	65.4 (24.9)	49.4 (24.6)	72.4 (22.1)	73.5 (18.6)
High beauty	82.3 (18.2)	22.8 (25.6)	76.2 (20.6)	54.7 (29.6)	77.4 (18.6)	73.5 (20.7)

The correlations were again first calculated for each individual participant, and then averaged over participants (see **Table 3** for the correlation matrix). The correlations revealed a strong positive association between liking and beauty (*r* = 0.76), and moderately strong correlations between perceived sadness and movingness (*r* = 0.49), and perceived beauty and movingness (*r* = 0.55). Although every attempt was made to manipulate levels of perceived beauty and sadness as independently as possible, the two concepts were still positively correlated to a limited extent (*r* = 0.25).

**Table 3:** Pearson correlations coefficients between ratings of perceived emotion, beauty, and liking.

	Liking	Beauty	Sad	Moving	Tender	Peaceful	Scary
Beauty	0.76						
Sad	0.16	0.25					
Moving	0.45	0.55	0.49				
Tender	0.38	0.48	-0.09	0.33			
Peaceful	0.36	0.50	0.04	0.38	0.70		
Scary	-0.36	-0.50	-0.02	-0.32	-0.56	-0.54	
Happy	0.11	0.07	-0.46	-0.18	0.23	0.09	-0.28

*The correlations were first calculated within each participant, and then averaged across participants.*

#### Mediation Analysis

The same multilevel mediation analysis method as used in Experiment 1 was used to analyze the data from the current experiment. First, we tested the hypothesis that perceived movingness would mediate the effect of perceived sadness on liking ratings (Hypothesis A) in a similar manner as being moved mediated the effect of felt sadness on liking in Experiment 1. The total effect of perceived sadness on liking was not quite significant (path c; β = 0.091, *t* = 2.08). However, the effect of perceived sadness on perceived movingness (path a; β = 0.47, *t* = 8.16), and the effect of perceived movingness on liking (path b; β = 0.43, *t* = 6.56) were significant. When perceived movingness was controlled for, the direct effect of perceived sadness on liking became significantly negative (path c′; β = -0.13, *t* = -2.62). The estimated indirect effect of perceived sadness on liking (mediated by perceived movingness) was 0.22 (95% CI [0.14; 0.31]); somewhat smaller than in Experiment 1, but still notable.

Next, we investigated the competing hypothesis (Hypothesis B) that – instead of movingness – perceived beauty would mediate the effect of perceived sadness on liking. As in the first mediation analysis, the total effect of perceived sadness on liking was not quite significant (path c; β = 0.086, *t* = 2.08). The effect of perceived sadness on beauty (path a; β = 0.15, *t* = 4.69) and the effect of beauty on liking (path b; β = 0.75, *t* = 11.35) were both significant. When perceived beauty is controlled for, the direct effect of perceived sadness on liking becomes negative (but non-significant; path c′; β = -0.033, *t* = -0.93). The estimated indirect effect of perceived sadness on liking (mediated by beauty) was 0.12 (95% CI [0.07; 0.17]); half the magnitude of the indirect effect mediated by movingness.

Finally, we investigated whether perceived movingness would also mediate the effect of perceived sadness on perceived beauty. The total effect of perceived sadness on beauty was significant (path c; β = 0.16, *t* = 4.99). The effect of perceived sadness on movingness (path a; β = 0.47, *t* = 8.07) and the effect of movingness on beauty (path b; β = 0.50, *t* = 8.37) were also significant. Again, when perceived movingness is controlled for, the direct effect of perceived sadness on beauty becomes negative (but non-significant; path c′; β = -0.068, *t* = -1.30). The estimated indirect effect of perceived sadness on beauty was 0.24 (95% CI [0.16; 0.33]).

### Discussion

The musical stimuli selected for Experiment 2 were intended to represent independently varying levels of sadness and beauty (high, medium, and low levels of both in a factorial design). However, the two concepts were still positively correlated (*r* = 0.25), albeit the correlation was considerably smaller than that reported in a previous study where the selection of stimuli was based on other criteria (*r* = 0.59; Eerola and Vuoskoski, 2011). The mean ratings displayed in **Table 2** indicate that the correlation may have been driven by the highly sad excerpts, as they exhibited less variance in terms of beauty compared to the other types of excerpts. Indeed, finding examples that would be highly sad but low in beauty proved to be especially difficult – at least when using Western film music as the stimulus material. This difficulty may be related to an inherent association between perceived sadness and beauty, or to stylistic conventions that are used in the Western music tradition to convey sadness. Future studies on the topic should strive to use musical materials from more varied cultural settings in order to further elucidate this issue.

In order to use a sufficiently large set of stimuli where levels of sadness and beauty could be varied systematically, the decision was taken to measure perceived rather than felt emotions. Perceived emotions can be reliably measured using very short excerpts (even as short as 1–5 s; Al’tman et al., 2000; Bigand et al., 2005), while a review by Eerola and Vuoskoski (2012) found that the median stimulus duration in studies investigating music-induced (felt) emotions was 90 s. This suggests that the induction of emotion (and sufficiently accurate introspection) is typically thought to require considerably more time than emotion recognition/perception. Thus, instead of investigating *feelings* of sadness and being moved, Experiment 2 measured the sad and moving qualities perceived in the music stimuli. This might partly explain why the mediation effect was slightly weaker than in Experiment 1 (although the selection of musical stimuli is likely to have played a role as well). However, it should be noted that perceived emotional qualities can directly lead to emotion induction (e.g., through emotional contagion; Juslin and Västfjäll, 2008). Indeed, significant overlap has been shown to exist between perceived and induced emotions (e.g., Kallinen and Ravaja, 2006; Schubert, 2013), and felt emotions that diverge from the perceived emotional expression of the music are typically driven by mechanisms such as episodic memories and evaluative conditioning (Juslin and Västfjäll, 2008; Juslin, 2013), which are typically associated with familiar music. As the present experiment used unfamiliar, experimenter-selected stimuli (familiarity ratings were collected by Eerola and Vuoskoski, 2011), it could be expected that the ratings of perceived emotion would not greatly diverge from hypothetical ratings of felt emotion.

We set out to test two competing hypotheses: (A) that movingness would mediate the effect of perceived sadness on liking, or (B) that perceived beauty would mediate the effect of sadness on liking. The results of the multilevel mediation analyses suggested that both perceived movingness and beauty may mediate the effect of perceived sadness on liking. However, the indirect effect via movingness was twice the magnitude of that via beauty, suggesting that perceived movingness provides a better account for the link between perceived sadness and liking. This interpretation is further supported by the fact that the positive association between perceived sadness and beauty was entirely mediated by perceived movingness.

## General Discussion

Our findings suggest that the pleasure drawn from sad music – similarly to the enjoyment of sad films – is mediated by being moved. In both experiments, the initial positive relationships between felt and perceived sadness and liking were entirely mediated by feelings and percepts of movingness. Our findings are in line with previous studies of music-induced sadness that have associated more intensely felt sadness with greater enjoyment (e.g., Vuoskoski et al., 2012; Eerola et al., 2016). However, we have provided new evidence of the significant role of being moved in this relationship. Our findings regarding the contribution of trait empathy to the feelings of sadness and being moved evoked by sad music are highly compatible with the notion of ‘being moved’ as a socially significant emotion that activates the value of social bonds and prosocial behavior (see Menninghaus et al., 2015).

But is there a musical equivalent for the pro-social features that characterize non-musical instances of being moved (e.g., Menninghaus et al., 2015; Wassiliwizky et al., 2015)? A growing body of research has established the significance of social cognition not only in music-making (e.g., Phillips-Silver and Keller, 2012; Moran, 2014), but also in music perception (Aucouturier and Canonne, 2017). A recent study by Aucouturier and Canonne (2017) showed that listeners could accurately decode social intentions (varying in the degree of affiliation and control) from improvised musical interactions, demonstrating that music can be perceived in terms of social relations between real and/or virtual agents. Moreover, certain musical features, namely harmonic and temporal coordination, were causally associated with the affiliation and control dimensions of social behavior (respectively). In addition to establishing that music can directly communicate social relational intentions, these findings are compatible with theoretical accounts proposing that music can be perceived and experienced as narratives of virtual persons ‘inhabiting’ the musical environment (e.g., Levinson, 2006). Thus, it is plausible that those pieces of sad music that listeners find particularly moving are perceived and experienced as communicating pro-social intentions, and may even afford a form of (para)social engagement – empathy – for the listener.

This interpretation is congruent with the finding that the particular subscales of trait empathy (namely Fantasy and Empathic Concern) that were associated with feelings of sadness and being moved in the present study, are specifically those that tap into the tendencies to imaginatively transpose oneself into the feelings of fictitious characters, and to engage in other-oriented, compassionate empathy and helping behavior (e.g., Davis, 1983; Eisenberg and Fabes, 1990). Furthermore, the association between trait empathy and being moved provides further support and explanation for previous findings that have linked trait empathy with the enjoyment of sad music (e.g., Garrido and Schubert, 2011; Vuoskoski et al., 2012; Taruffi and Koelsch, 2014; Eerola et al., 2016). The findings of the present study suggest that trait empathy contributes directly to the intensity of felt sadness and movingness (and thus enjoyment), but does not modulate the relationships between felt sadness, being moved, and liking.

Our findings and conclusions are subject to certain limitations. As the majority of the participants in Experiment 1 were non-native English speakers, it is possible that there were differences in the participants’ understanding of the rating scale labels. Furthermore, since the majority of the participants in both experiments were female, our findings may not be equally generalizable to men (although most studies that have investigated the role of gender in music-related emotional processing have failed to find significant differences; see Eerola and Vuoskoski, 2012). By default, online experiments have less control in terms of audio quality and participant concentration, but a number of studies have also suggested that the inherent variability present in online studies is linked to clear advantages (such as a wider sample pool; Gosling et al., 2004; Kraut et al., 2004). However, the close similarities between the findings of Experiments 1 and 2 (with Experiment 2 using a more controlled setting albeit a smaller sample of participants), suggest that the pattern of mediation remains consistent despite the differences in language, setting, and musical material.

The present study has not exhaustively explored feelings of ‘being moved’ in the context of music-induced sadness, or its interactions with aesthetic appreciation or liking. It has, however, highlighted the significance of the phenomenon, and opened new avenues for further investigation. Future studies on music-induced feelings of being moved should investigate the physiological responses commonly associated with feelings of being moved (e.g., chills and skin conductance; Benedek and Kaernbach, 2011; Wassiliwizky et al., 2015) as well as the musical aspects that are conducive to being moved. The latter could entail exploring the perceived social intentions in music and their underlying acoustic features (following the work of Aucouturier and Canonne, 2017), or manipulating the aesthetically pleasing qualities of music (sad music in particular). This could help to clarify whether ‘being moved’ in the context of music listening would be better conceptualized as a social emotion (cf. Menninghaus et al., 2015), or as an aesthetic response (as proposed by Konecni, 2005).

Our findings have certainly highlighted the difficulty of disentangling perceived sadness and perceived beauty when using existing musical material. Although we allow that the conceptual distinction between liking and perceived beauty may be somewhat artificial (and that significant overlap most likely exists between the two), we nevertheless showed that perceived movingness mediated the effect of perceived sadness on both liking and perceived beauty. Furthermore, when two alternative paths from perceived sadness to liking were considered – one via movingness and the other via beauty – the estimated indirect effect via movingness was double the magnitude of that via beauty. Thus, we argue that being moved may provide a more persuasive account of the paradox of “pleasurable sadness” than aesthetic appreciation. Contrary to Juslin’s (2013) statement, we argue that felt sadness does in fact contribute to the enjoyment of sadness-inducing music by directly intensifying feelings of being moved, and that the sadness *per se* can thus be considered a source of pleasure.

## Author Contributions

JV conceived the study idea, and was responsible for the study design, carrying out the experiments, analyzing the data, and writing the article. TE contributed significantly to the study design, data analysis, and writing the article.

## Conflict of Interest Statement

The authors declare that the research was conducted in the absence of any commercial or financial relationships that could be construed as a potential conflict of interest.
