# Supplementary material for: The Pleasure Evoked by Sad Music Is Mediated by Feelings of Being Moved
Source: Front Psychol. 2017 Mar 21;8:439. doi: 10.3389/fpsyg.2017.00439 (PMC5359245; doi:10.3389/fpsyg.2017.00439)
Supplement: Supplementary file 1 [file List_of_Excerpts.PDF]

Supplementary material: List of excerpts used in Experiment 2

Stimulus name	Soundtrack name	Track	Min:Sec	Stimulus number	Mean beauty	Mean sadness	Beauty level	Sadness level
LSLB1	Batman Returns	5	00:09–00:25	011*	25.69	13.14	Low	Low
LSLB2	The English Patient	8	01:35–01:57	066*	40.24	26.48	Low	Low
LSLB3	Running Scared	8	00:20-00:35	095†	30.63	28.60	Low	Low
LSMB1	Dances with Wolves	4	01:31–01:48	043*	69.08	13.22	Medium	Low
LSMB2	Man of Galilee CD1	2	00:19–00:42	056*	59.80	10.19	Medium	Low
LSMB3	Oliver Twist	7	01:30–01:46	080*	61.87	23.87	Medium	Low
LSHB1	Pride & Prejudice	1	00:10–00:26	042*	87.65	18.11	High	Low
LSHB2	Pride & Prejudice	12	00:01–00:15	044*	83.01	32.41	High	Low
LSHB3	The Missing	3	00:24-00:49	066†	76.34	17.74	High	Low
MSLB1	Shakespeare in Love	11	00:21-00:36	117†	46.42	43.64	Low	Medium
MSLB2	Nostradamus	15	02:48-03:05	120†	51.14	60.01	Low	Medium
MSLB3	Gladiator	8	00:22-00:37	–	53.05	48.90	Low	Medium
MSMB1	Shakespeare in Love	3	00:59–01:17	039*	67.80	44.34	Medium	Medium
MSMB2	The English Patient	8	00:38-00:57	174†	68.13	55.20	Medium	Medium
MSMB3	Oliver Twist	6	00:35-00:50	–	60.22	48.53	Medium	Medium
MSHB1	Pride & Prejudice	9	00:01–00:21	054*	90.26	47.95	High	Medium
MSHB2	The English Patient	7	00:00–00:31	040*	69.50	55.45	High	Medium
MSHB3	Band of Brothers	7	00:17-00:34	–	68.92	60.80	High	Medium
HSLB1	Road to Perdition	16	00:17-00:32	037†	66.35	63.32	Low	High
HSLB2	Band of Brothers	4	00:24-00:44	118†	61.71	67.72	Low	High
HSLB3	Shakespeare in Love	9	00:00-00:22	150†	68.92	73.81	Low	High
HSMB1	Band of Brothers	12	01:09-01:26	034†	73.97	74.97	Medium	High
HSMB2	Running Scared	15	02:06-02:27	036†	71.36	74.10	Medium	High
HSMB3	Band of Brothers	18	00:40-01:00	149†	71.94	71.40	Medium	High
HSHB1	The Four Feathers	10	01:15-01:34	031†	82.06	70.62	High	High
HSHB2	The Portrait of a Lady	9	00:00–00:22	033*	75.88	79.15	High	High
HSHB3	Psycho	3	00:58-01:24	038†	74.18	70.74	High	High

* Stimulus number in the set of 110; Eerola & Vuoskoski, 2011
† Stimulus number in the set of 360; Eerola & Vuoskoski, 2011
